# Iguratimod Attenuates Macrophage Polarization and Antibody-Mediated Rejection After Renal Transplant by Regulating KLF4

**DOI:** 10.3389/fphar.2022.865363

**Published:** 2022-05-09

**Authors:** Zhou Hang, Jintao Wei, Ming Zheng, Zeping Gui, Hao Chen, Li Sun, Shuang Fei, Zhijian Han, Jun Tao, Zijie Wang, Ruoyun Tan, Min Gu

**Affiliations:** ^1^ Department of Urology, The Second Affiliated Hospital of Nanjing Medical University, Nanjing, China; ^2^ Department of Emergency Medicine, The Second Affiliated Hospital, Zhejiang University School of Medicine, Hangzhou, China; ^3^ Department of Urology, The First Affiliated Hospital of Nanjing Medical University, Nanjing, China

**Keywords:** macrophage polarization, antibody-mediated rejection, KLF4, iguratimod, kidney transplant

## Abstract

**Background:** This study aimed to explore the effect and mechanism of iguratimod (IGT) on M1 macrophage polarization and antibody-mediated rejection (ABMR) after renal transplant.

**Methods:** Bioinformatics analysis was performed using three public databases derived from the GEO database. Sprague–Dawley (SD) rats were pre-sensitized with donors of Wistar rats in skin transplantation and a rat renal transplant ABMR model was established from the donors to skin pre-sensitized recipients. Subsequently, IGT was treated on the ABMR model. Routine staining and immunofluorescence (IF) staining were performed to observe the pathological changes in each group and flow cytometry was performed to detect the changes of DSA titers in peripheral blood. In addition, bone-marrow-derived macrophage (BMDM) was extracted and interfered with IGT to explore the effect of IGT *in vivo*. PCR, IF staining, and Western blot were used to detect the expression of related genes and proteins.

**Results:** Bioinformatics analysis revealed that several immune cells were significantly infiltrated in the ABMR allograft, while M1 macrophage was noticed with the most significance. Results of IF staining and PCR proved the findings of the bioinformatics analysis. Based on this, IGT was observed to significantly attenuate the degree of peritubular capillary vasculitis and arteriolitis in the rat renal transplant ABMR model, whereas it decreases the expression of C4d and reduces the titer of DSA. Results *in vitro* suggested that M1 macrophage-related transcripts and proteins were significantly reduced by the treatment of IGT in a dose- and time-dependent manner. Furthermore, IGT intervention could remarkably decrease the expression of KLF4.

**Conclusion:** Polarization of M1 macrophages may aggravate ABMR after renal transplant by promoting DSA-mediated endothelial cell injury, and IGT may attenuate the pathogenesis of ABMR by targeting KLF4.

## Introduction

Allograft rejection after kidney transplantation is the main factor affecting the long-term survival of renal allograft (Gerlach et al., 2017; Wang et al., 2017; Nunes-Dos-Santos et al., 2020). According to its mechanism, rejection episodes following renal transplant can be classified into T-cell-mediated rejection (TCMR) and humoral rejection (Rodríguez-Perálvarez et al., 2017; Haas, 2018). At present, most immunosuppressive drugs on the market are aimed at T-cell-mediated rejection. However, an increasing number of studies have noticed the profound effect of antibody-mediated rejection (ABMR) on the long-term allograft function, which was considered as one of crucial factors contributing to graft loss (Loupy and Lefaucheur, 2018; Louis et al., 2019).

Previous studies have reported that donor endothelial cells are the initial target of *de novo* antibodies (DSA) (Piotti et al., 2014; Valenzuela et al., 2014; Cardinal et al., 2017). As antibodies bind to the corresponding antigens of endothelial cells, inflammatory damage to the vascular endothelium of the graft was caused through complement-dependent and independent pathways (Djamali et al., 2014; Horwitz et al., 2019). In the complement-dependent pathway, C4d has been considered a specific diagnostic marker of ABMR by the Banff diagnostic criteria, and complement inhibitors targeting C5 have been used to treat ABMR (Jordan et al., 2020; Patel et al., 2021). In complement-independent pathway studies, various types of immune cells, including NK cells, macrophages, T and B lymphocytes, and dendritic cells, were observed to be involved in chronic ABMR (Koenig et al., 2019). Moreover, recent studies have found that increasing the depletion of M1 macrophages can significantly reduce the occurrence of tissue damage and rejection (Cheng et al., 2015; Dai et al., 2020).

Macrophages can be divided into M1 macrophages (promoting inflammatory response) and M2 macrophages (promoting cell proliferation and mediating tissue repair) (Shapouri-Moghaddam et al., 2018; Orecchioni et al., 2019). Under different conditions of immune microenvironments and stimulating factors, macrophages can polarize into M1 or M2 macrophages, which play an important role in maintaining the homeostasis of immune microenvironments by secreting different cytokines (Mills, 2012). M1 macrophages exert pro-inflammatory and anti-tumor biological effects mainly by secreting cytokines such as tumor necrosis factor-α (TNF-α) and interleukin1-beta (IL-1β) (Jakubzick et al., 2017). A bioinformatics study containing 1,697 biopsy samples found that M1 macrophage-associated gene scores were significantly elevated in ABMR biopsies and were strongly associated with poor prognosis (Azad et al., 2018). In addition, some studies have shown that pro-inflammatory cytokines such as TNF-α and IL-1β, which are significantly increased in the progression of pathological injury, are mainly secreted by macrophages (Riquelme et al., 2013). Therefore, it is strongly believed that the secretion of pro-inflammatory factors after the polarization of macrophages may be related to the progression of ABMR.

The Kruppel-like factor (KLF) family is a class of DNA-bound transcription regulatory factors of zinc-finger proteins, which play an important regulatory role in various physiological functions (Takahashi and Yamanaka, 2006). It has been confirmed that KLF4 deficiency can lead to the activation of NF-κB transcriptional activity in macrophages, promoting the polarization of M1 macrophages and significantly enhancing the inflammatory function of M1 macrophages (Liao et al., 2011a). Recently, it was found that the lack of KLF4 can significantly increase the infiltration of M1 macrophages in kidney tissue and accelerate the progression of chronic nephritis in the mouse model of chronic kidney injury, ultimately resulting in end-stage renal disease and renal fibrosis (Wen et al., 2019). These results suggest that KLF4 may be involved in the M1 polarization in ABMR after kidney transplantation.

Iguratimod (IGT) is a small molecule anti-inflammatory drug, which has been widely used in the treatment of rheumatoid arthritis because of its efficient and safe anti-cytokine-mediated inflammatory response (Hara et al., 2014; Ishikawa and Ishikawa, 2019). Previous studies have shown that IGT can play an important role in anti-inflammatory and immune regulation by inhibiting the synthesis of prostaglandin E2, TNF-α, and interleukin-17 (IL-17) (Liu et al., 2021). Studies have shown that IGT can delay kidney injury in mouse models of lupus nephropathy by inhibiting antibody-mediated immune response (Yan et al., 2014; Zhao et al., 2019). More importantly, our previous clinical study on the application of IGT in transplantation reported that the combination of IGU and conventional immunosuppressants can reduce the probability of rejection after transplantation (Tao et al., 2021). Therefore, whether IGT could attenuate the progression of ABMR still remains unknown.

From what has been discussed previously, we hypothesized that IGT may inhibit the polarization of M1-type macrophages by upregulating the expression of KLF4, reduce the secretion of TNF-α cytokines, and consequently alleviate DSA-mediated allograft endothelial injury and the progression of ABMR after renal transplantation.

## Methods and Materials

### Ethnic Statement

The research protocol meets the ethical standards of the Declaration of Helsinki and Istanbul. The research protocol involving human kidney tissues was approved by the local ethics committee of the First Affiliated Hospital of Nanjing Medical University (2016-SR-029). Written informed consent was obtained from all transplant recipients and nephrectomy patients.

### Bioinformatics Analysis

The series of matrix files of GSE36059, GSE98320, and GSE124203 were downloaded from the GEO database (https://www.ncbi.nlm.nih.gov/geo) and merged into a metadata cohort after removing the batch effect by the “SVA” package of R software. The “limma” package was used to identify differentially expressed genes. To quantify the relative proportions of infiltrating immune cells, a bioinformatics algorithm was used to calculate immune cell infiltrations by the CIBERSORT R script. The putative abundance of immune cells was estimated using a reference set with 22 types of immune cell subtypes (LM22) with 1,000 permutations (Ali et al., 2016). We used Spearman’s rank correlation analysis performed by the R package “corrplot” and “ggplot2” when exploring the association.

### Animals

Adult male Sprague–Dawley (SD) and Wistar rats were procured from Charles River Laboratories (Beijing, China). Adult male Balb/c mice were procured from the Animal Core Facility of Nanjing Medical University. The animal center provided the animals with clean tap water and standard feedstuff. All animal handling procedures followed guidelines issued by the US National Institutes of Health and animal ethics established by Nanjing Medical University.

### Establishment of Rat Renal Transplant ABMR Model

We pre-sensitized recipient SD rats with donor Wistar rats by skin transplantation and constructed an ABMR model by abdominal ectopic kidney transplantation from donors to skin pre-sensitized recipients. Based on ABMR models, we gave a gavage of IGT (15 mg/kg) every morning and evening after surgery to explore the effect of IGT on ABMR. Detailed procedures are described in previous studies (Cheng, 2019).

### Isolation, Culture, and Treatment of Bone-Marrow-Derived Macrophage

Bone marrow suspension was obtained by rinsing the bone marrow cavity of the femur and tibia of Balb/c mice. A monocyte separator (LTS1092PK-200, tbdscience) was used to isolate monocytes from the bone marrow suspension. Isolated monocytes were cultured with DMEM (01-05201ACS, Biological Industries) containing 10% FBS (04-001-1ACS, Biological Industries), 1% penicillin-streptomycin, and 30 ng/ml M-CSF (CB34, novoprotein). The new medium was replaced 3 days later. The cells matured 7 days after the extraction and could be used for downstream experiments. BMDMs were stimulated with 50 ng/ml LPS (00-4976-93, Invitrogen) and 10 ng/ml IFN-γ (CM41, Selleck) to promote M1 polarization. To explore the effect of IGT *in vitro*, cells were treated for 24 h with IGT (10 ng/ml, S5648, Selleck).

### Cell Counting Kit-8 Analysis

The cell suspension was prepared and the cell number was counted. Planking cells (about 1–2 × 10^4^) were inoculated into a 96-well plate. After incubation at 37 °C for 4 h, 10uL of CCK8 (Beyotime, Shanghai, China) was added and cells were cultured for 0.5–4 h. It is recommended to use dual wavelengths to determine the absorbance, with a detection wavelength of 450–490 nm and a reference wavelength of 600–650 nm (Hou et al., 2007).

### RNA Isolation and Quantitative Real-Time PCR

RNA was isolated from cells or tissues using the commercial RNA Isolation Kit (RC112-01, Vazyme). cDNA was synthesized with a reverse transcription kit (R333-01, Vazyme). Quantitative real-time PCR was performed with a SYBR Green PCR kit (Q341-AA, Vazyme). The specific primers used were as follows: CD68: 5′‐TGT​CTG​ATC​TTG​CTA​GGA​CCG‐3′ (F),5′-GAGAGTAACGGCCTTTTTGTGA‐3′ (R); CD86: 5′‐TGTTTCCGTGGAGACGCAAG‐3′(F),5′-TTGAGCCTTTGTAAATGGGCA‐3′ (R); NOS2: 5′‐GTT​CTC​AGC​CCA​ACA​ATA​CAA​GA‐3′ (F),5′- GTG​GAC​GGG​TCG​ATG​TCA​C‐3′ (R); CD206: 5′‐CTC​TGT​TCA​GCT​ATT​GGA​CGC‐3′ (F),5′- CGG​AAT​TTC​TGG​GAT​TCA​GCT​TC‐3′ (R); IL-1β: 5′‐TTC​AGG​CAG​GCA​GTA​TCA​CTC‐3′ (F),5′-GAAGGTCCACGGGAAAGACAC.‐3′ (R); IL-6: 5′‐TAG​TCC​TTC​CTA​CCC​CAA​TTT​CC‐3′ (F),5′- TTG​GTC​CTT​AGC​CAC​TCC​TTC‐3′ (R); KLF4: 5′‐GTG​CCC​CGA​CTA​ACC​GTT​G‐3′ (F),5′- GTC​GTT​GAA​CTC​CTC​GGT​CT‐3′ (R); and GAPDH: 5′‐GAA​GGT​CGG​TGT​GAA​CGG​AT‐3′ (F),5′-CCCATTTGATGTTAGCGGGAT‐3′ (R). The primers were bought from Tsingke Biotechnology (Nanjing, China).

### Histology and Immunofluorescence Microscopy

Kidney tissue sections were stained with HE and PAS for the histopathological examination. Immunofluorescence microscopy was performed as previously described, using antibodies against C4d (HP8034, Hycult Biotec), CD68 (ab955, Abcam), iNOS (ab178945, Abcam), iNOS (ab49999, Abcam), F4/80 (28463-1-AP, proteintech), and KLF4 (1180-1-AP, proteintech) (Wang et al., 2017). Images from each stained slide were captured, and quantitative analyses of positive staining were carried out independently by two authors (Z Hang and M Zheng) under a light microscope equipped with a digital camera (Nikon, ECLIPSE 80i, Japan). The mean fluorescence intensity (MFI) of each image was calculated and mean relative abundance was obtained from MFIs of five representative images.

### Flow Cytometry

Flow cytometry was performed to identify macrophage polarization. We use fluorescein isothiocyanate (FITC)-conjugated anti-mouse iNOS (5201004082, Miltenyi Biotec) to identify M1 macrophages and phycoerythrin (PE)-conjugated anti-mouse F4/80 (5200809116, Miltenyi Biotec) to identify M0 macrophages according to the instructions.

### DSAs Examination

We co-cultured the spleen lymphocytes of normal Wistar rats with the serum of the rats to be tested. The cells were incubated with PE-conjugated anti-rat IgG (GB21302, Servicebio) after cleaning. The mean fluorescence intensity (MFI) of the cell suspension was measured by flow cytometry and the level of serum DSA was evaluated (Chandran et al., 2018; Zhao et al., 2018).

### Statistical Analysis

All analyses were independently repeated at least three times. All data were expressed as the mean ± SD value determined by three independent experiments. One-way analysis of variance (ANOVA) was used for intergroup comparison. Multiple methods were compared using the Tukey test. The difference between the two groups was determined by a non-parametric test with regard to the limited sample size. *p* < 0.05 was considered statistically significant.

## Results

### M1 Macrophage Infiltration Increased Significantly in ABMR Allograft

**FIGURE 1:**
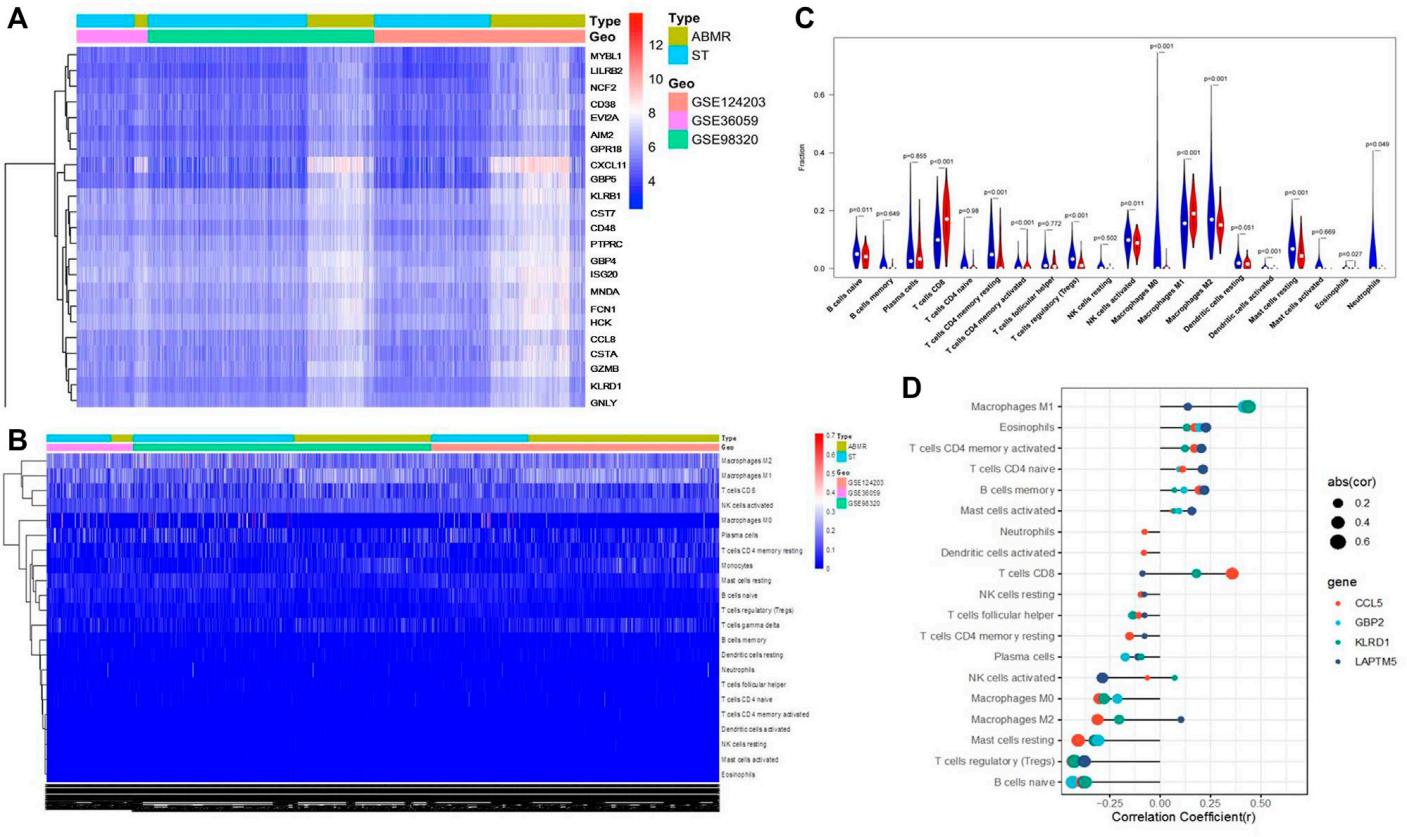
Bioinformatics analysis results of three public databases from the GEO database. **(A)** Heat map after the joint analysis of the three databases. **(B)** Cell distribution after the combined analysis of the three databases. **(C)** Analysis of immune cell infiltration in ABMR transplanted kidney tissue. **(D)** Correlation between immune cell infiltration and ABMR in renal transplantation.

The CIBERPORT algorithm was used to explore the immune infiltration in renal allograft specimens from three GEO databases (GSE124203, GSE36059, and GSE98320). Figures 1A,B show that macrophage polarization was significantly activated during the pathogenesis of ABMR in the pooled results. Moreover, it was found that macrophage infiltration, including macrophage M0, M1, and M2 types in allograft tissue with ABMR increased significantly (Figure 1C), while M1 macrophages were suggested as the most significant cells infiltrated in the progression of ABMR (Figure 1D), suggesting that M1 macrophages may play an important role in the pathogenesis of ABMR.

**FIGURE 2:**
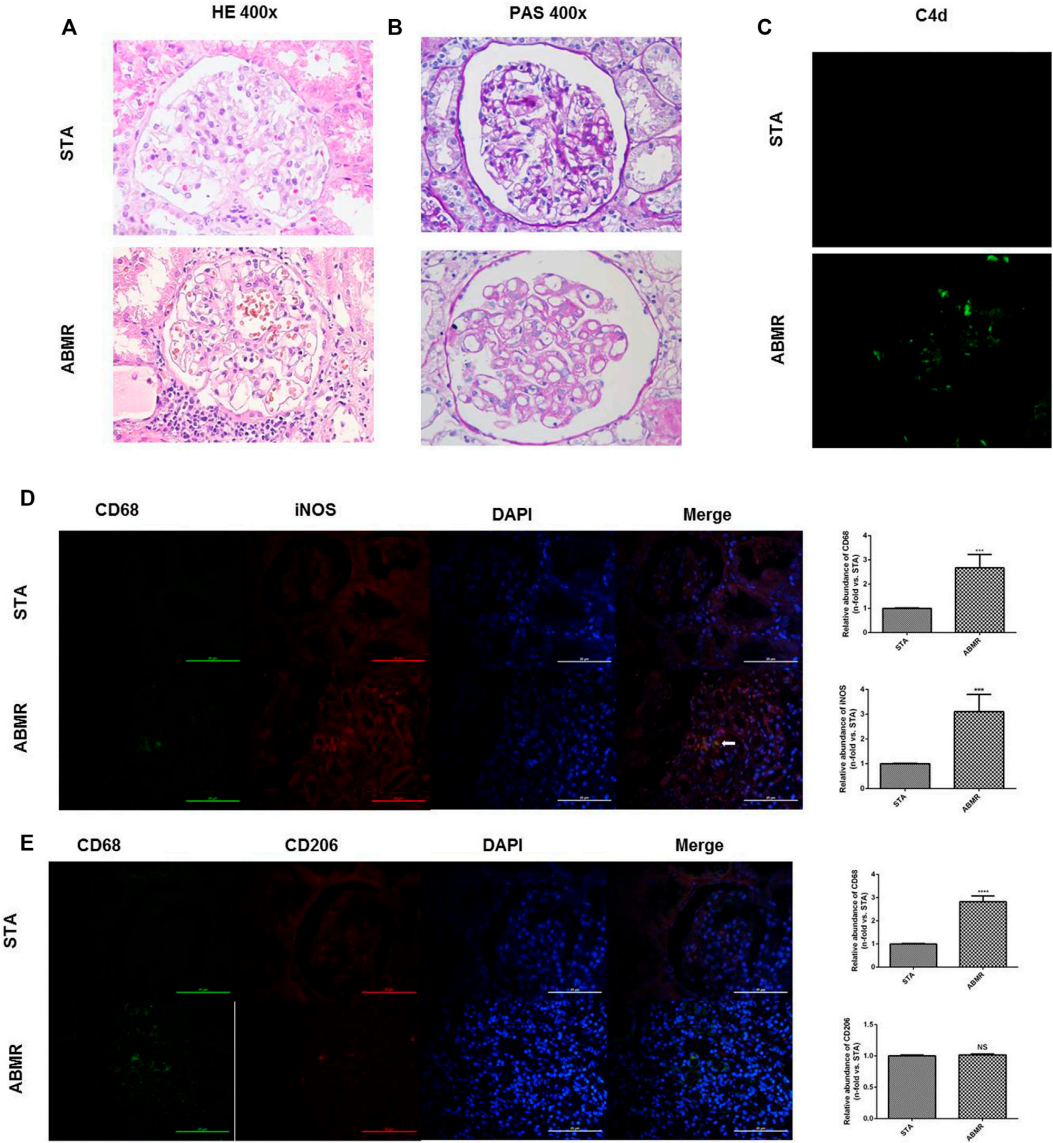
Determination of macrophage infiltration in human renal allograft samples. **(A–B)** Renal pathology in STA and ABMR patients (400×). **(C)** Representative images of C4d immunostaining in kidney sections from STA and ABMR patients (400×). **(D)** Double indirect fluorescence staining of CD68 and iNOS in ABMR and STA groups (400×). **(E)** Double indirect fluorescence staining of CD68 and CD206 in ABMR and STA groups (400×). ***p < 0.001 compared with the STA group; ****p < 0.0001 compared with the STA group; NS: no significant difference compared with the STA group; a non-parametric test was used for statistical analysis in this figure.

Significant renal injury and more C4d deposition in peritubular capillaries and glomeruli in the ABMR group when compared with the STA group were observed by histological studies and immunofluorescence microscopy (Figures 2A–C). Moreover, infiltration of M1 macrophages was increased significantly in ABMR allografts (Figure 2D), which was mainly observed around the area of glomerular and peritubular capillaries. However, no significant difference of M2 macrophages was observed between the two groups (Figure 2E).

### Polarization of M1 Macrophages Increased in Rat Renal Transplant ABMR Model

**FIGURE 3:**
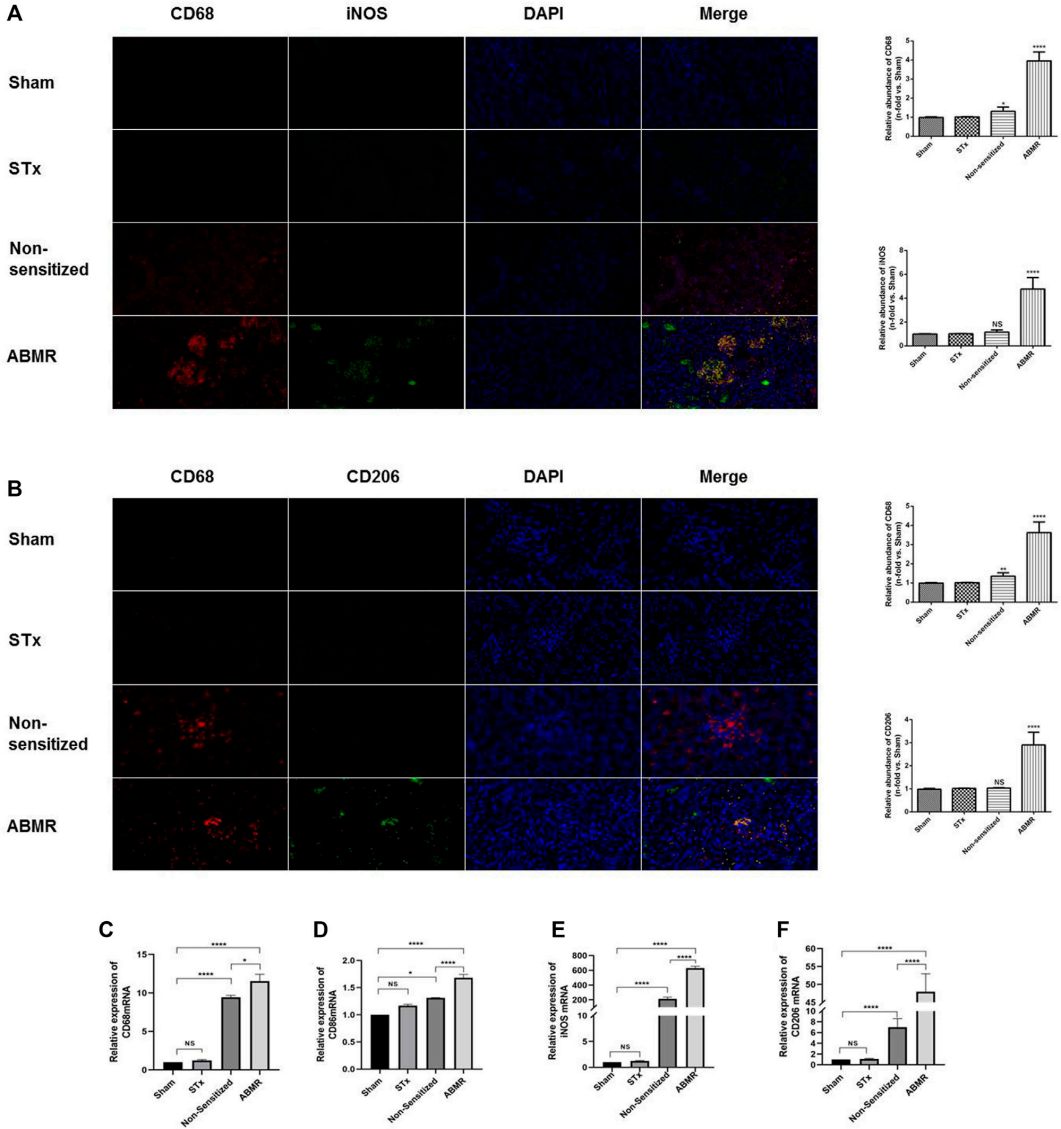
Determination of macrophage infiltration in renal allograft samples derived from the rat renal transplant ABMR model. **(A)** Double indirect fluorescence staining of CD68 and iNOS in the sham operation group (sham), skin transplant group (STx), non-sensitive kidney transplant group (non-sensitive), and ABMR group (ABMR) (200×). **(B)** Double indirect fluorescence staining of CD68 and iNOS in the sham group, STx group, non-sensitive group, and ABMR group (200×). **(C–F)** Renal mRNA expression of **(C)** CD68 **(D)** CD86 **(E)** iNOS, and **(F)** CD206 in the sham group, STx group, non-sensitive group, and ABMR group. *p < 0.05 compared with the sham group; **p < 0.01 compared with the sham group; ***p < 0.001 compared with the sham group; ****p < 0.0001 compared with the sham group; NS: no significant difference compared with the sham group; a non-parametric test was used for the statistical analysis in this figure.

We established a rat renal transplant ABMR model by skin pre-sensitization. Immunofluorescence microscopy revealed that iNOS, the specific transcription factor of M1 macrophages, was highly expressed in the ABMR group compared with other groups (Figure 3A). The expression of CD206 in the ABMR group was also increased (Figure 3B). Furthermore, compared with the non-pre-sensitized group, CD206, CD68, CD86, and iNOS increased in the ABMR group, but CD86 and iNOS were more significant (Figures 3C–F).

### IGT Attenuated the Progression of ABMR and M1 Macrophage Polarization in Rat Model

**FIGURE 4:**
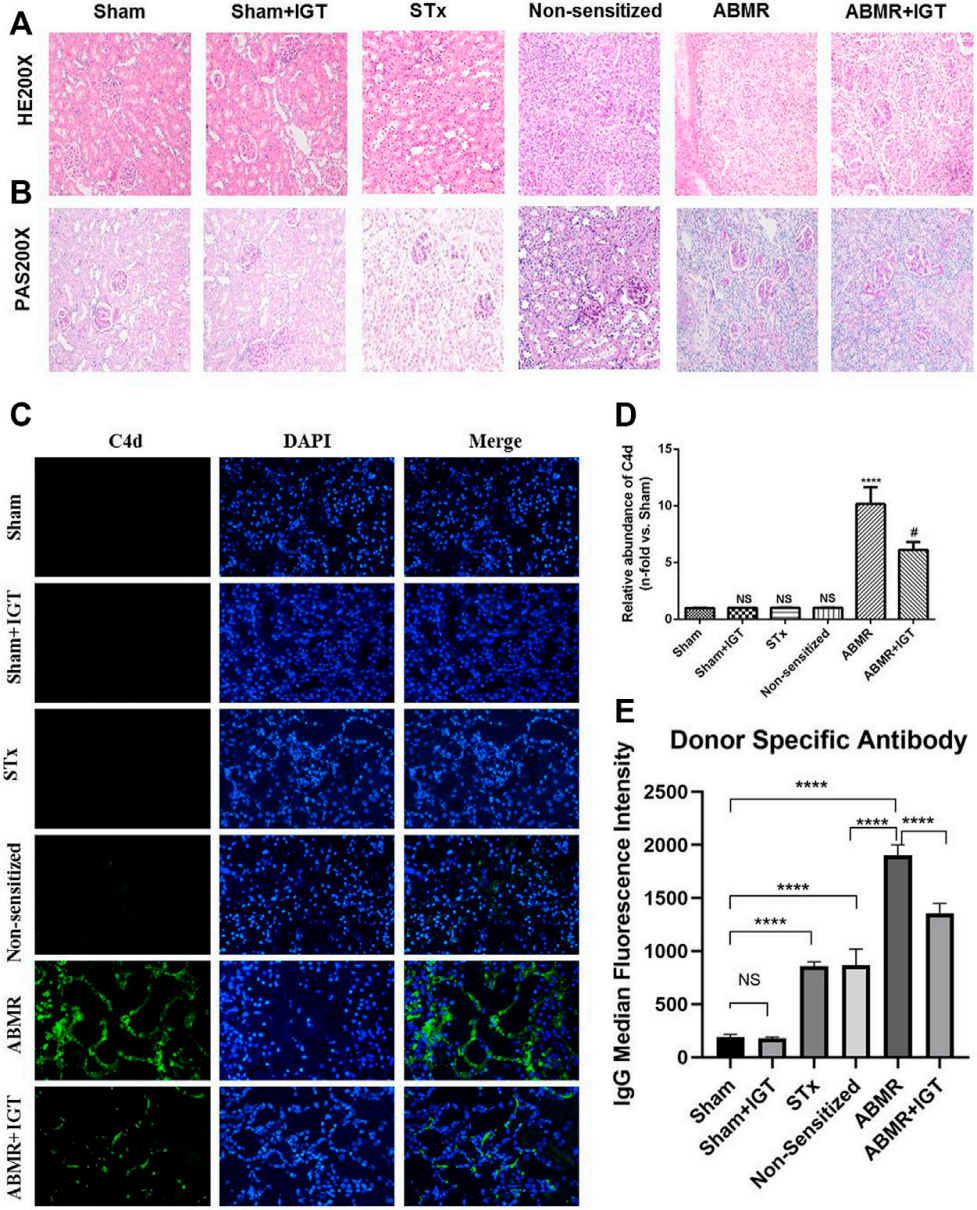
Effect of iguratimod intervention in the rat renal transplant ABMR model. **(A–B)** Representative images of renal pathology in sham, sham + IGT, STx, non-sensitive, ABMR, and ABMR + IGT groups (400×). **(C)** Indirect fluorescence staining of C4d in sham, sham + IGT, STx, non-sensitive, ABMR, and ABMR + IGT groups (400×). **(D)** Quantitative analysis of C4d immunostaining in each group. **(E)** DSA examination of sham, sham + IGT, STx, non-sensitive, ABMR, and ABMR + IGT groups. ****p < 0.0001 compared with the sham group; #*p* < 0.001 compared with the ABMR group; the non-parametric test was used for the statistical analysis in this figure.

HE and PAS staining showed significant peritubular capillary inflammation and glomerulitis in the ABMR group. Compared with the ABMR group, the inflammation in ABMR + IGT group was significantly relieved (Figures 4A,B). IGT can reduce the deposition of C4d and DSA which further confirms the effect of IGT (Figures 4C,D).

**FIGURE 5:**
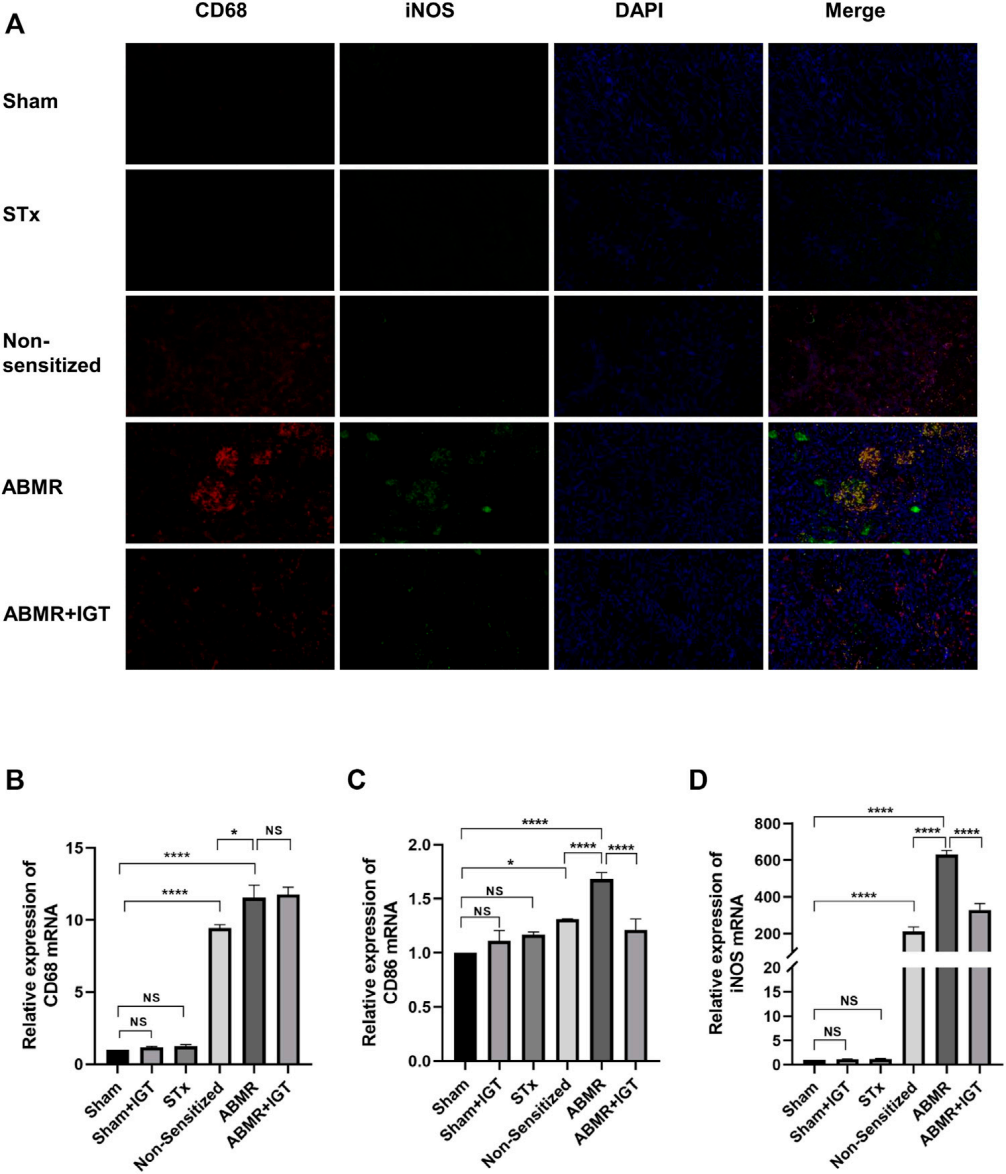
Determination of macrophage infiltration after intervention of IGT in the rat model. **(A)** Double indirect fluorescence staining of CD68 and iNOS in sham, sham + IGT, STx, non-sensitive, ABMR, and ABMR + IGT groups (400×). **(B–D)** Renal mRNA expression of **(B)** CD68 **(C)** CD86, and **(D)** iNOS in the sham, STx, non-sensitive, and ABMR groups. The non-parametric test was used for statistical analysis in this figure.

Immunofluorescence microscopy revealed remarkably high expression of iNOS in the ABMR group (Figure 5A). After the treatment of IGT, the positive area of iNOS in the allograft was dramatically decreased (Figure 5A). Results of the quantitative real-time PCR also indicated that IGT could downregulate the expression of CD86 and iNOS in the rat allograft kidney (Figures 5B–D). Therefore, the remission of ABMR progression by IGT may be correlated with the inhibition of M1 macrophage polarization.

### IGT Inhibited M1 Macrophage Polarization *in Vitro*

**FIGURE 6:**
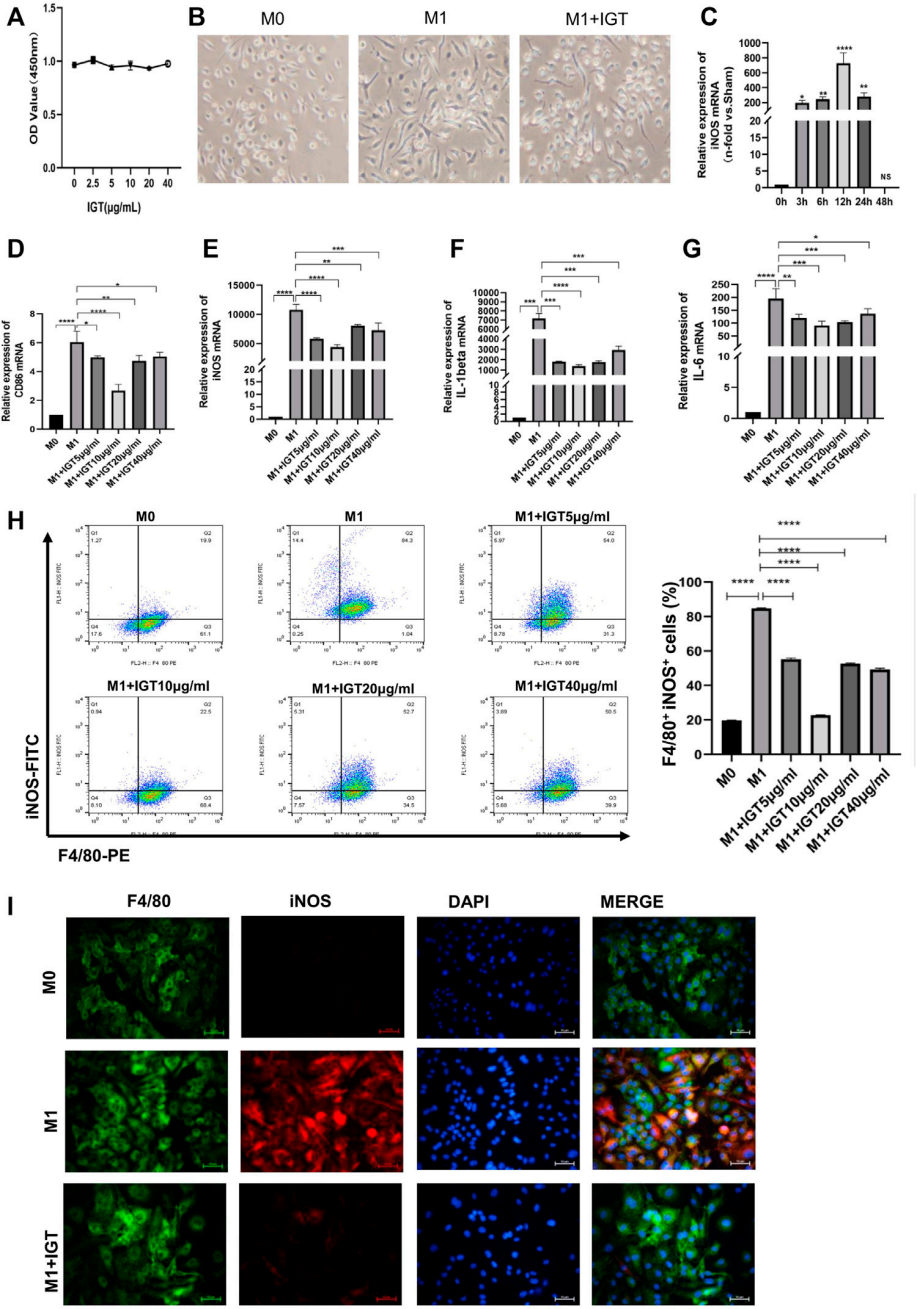
IGT-inhibited M1 macrophage polarization *in vitro.* **(A)** CCK8 assay of IGT intervention with BMDM. **(B)** Representative images of different macrophages under a light microscope (400×). **(C)** mRNA expression of iNOS in BMDM after the time-dependent intervention of IGT. **(D–G)** mRNA expression of CD86, iNOS, IL1-β, and IL6 in BMDM after concentration-dependent intervention of IGT. **(H)** Flow cytometry for F4/80 and iNOS in BMDM after concentration-dependent intervention of IGT. **(I)** Double indirect fluorescence staining of F4/80 and iNOS in M0 (M0), M1 (M1), and M1 macrophages after IGT intervention (M1+IGT). The non-parametric test was used for statistical analysis in this figure.

We used CCK8 to detect the toxicity of IGT and little effect has been noticed in the dose-dependent manner of IGT on cell proliferation (Figure 6A). Under a light microscope, the morphology of macrophages becomes thin during M1 polarization and IGT intervention can partially reverse the morphology of macrophages (Figure 6B). With the extension of IGT intervention, the change of iNOS at the transcription level reached the peak at 12 h and lost its effect after 48 h (Figure 6C). IGT of different concentrations (5, 10, 20, and 40 μg/ml) can inhibit macrophage polarization and reduce the secretion of pro-inflammatory factors, such as IL1-β and IL-6, at the transcription level (Figures 6D–G). This result was further confirmed by flow cytometry and immunofluorescence (Figures 6H,I). To further explore the effect of IGT on M2 macrophages, IGT intervention (10 μg/ml) was performed on M2 macrophages for 12 h. As shown in Supplementary Figure S1, no significant difference was observed in YM1, Fizz1, Arg-1, and MR mRNA between M2 macrophage with or without IGT (Supplementary Figure S1).

### KLF4 Was Involved in Macrophage Polarization Regulation in ABMR

**FIGURE 7:**
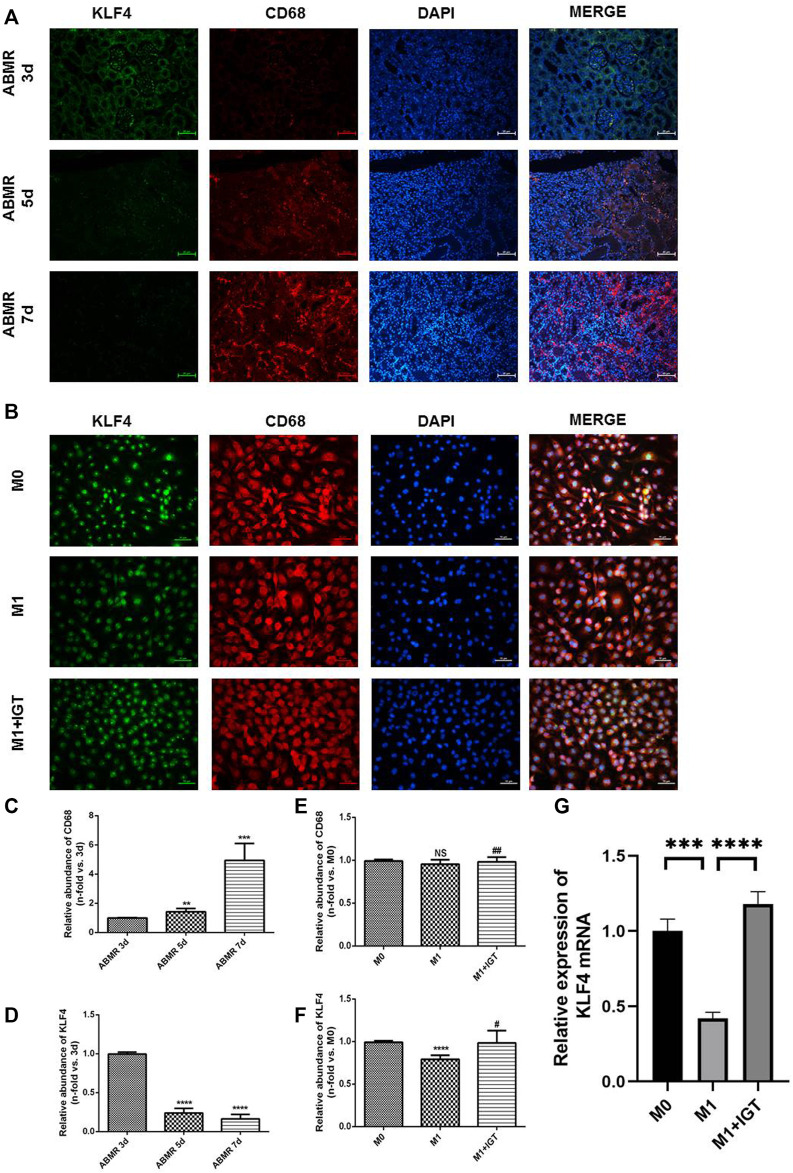
KLF4 was involved in macrophage polarization regulation in ABMR. **(A)** Double indirect fluorescence staining of CD68 and KLF4 in ABMR rat models. **(B)** Double indirect fluorescence staining of CD68 and KLF4 in M0, M1, and M1+IGT. **(C–D)** Quantitative analysis of CD68 **(C)** and KLF4 **(D)** in ABMR rat models. **(E–F)** Quantitative analysis of CD68 **(E)** and KLF4 **(F)** in cell culture. **(G)** mRNA expression of KLF4 in macrophages. **p < 0.01 compared with the control group; ***p < 0.001 compared with the control group; ****p < 0.0001 compared with the control group; #*p* < 0.05 compared with the M1 group; ##*p* > 0.05 compared with the M1 group; non-parametric test was used for statistical analysis in this figure.

Previous studies have noticed that KLF4 may be involved in the polarization of M1 macrophages, which could be served as a critical target for the modulation of macrophage polarization (Almatroodi et al., 2020; Wang et al., 2020; Qiu et al., 2021). Allograft from ABMR rats at different time points (3, 5, and 7 days) post-transplant were stained by indirect immunofluorescence. The degree of ABMR gradually increased, macrophage infiltration increased, and the expression of KLF4 in macrophages decreased in a time-dependent manner (Figure 7A). We also found that the expression of KLF4 mRNA and protein decreased significantly during the polarization of M1 macrophages. The expression of KLF4 mRNA and protein also increased significantly after the intervention of 10 μg/ml IGT (Figures 7B–G), suggesting that KLF4 may be involved in the process of IGT inhibiting the polarization of M1 macrophages.

## Discussion

In the present study, we reported that IGT could attenuate ABMR after kidney transplantation via regulating M1 macrophage polarization induced by the deficiency of the transcription factor KLF4. The roles of IGT are not only to block M1 macrophage polarization but to also largely reduce the manufacture of proinflammatory cytokines such as TNF-α and IL6 which are closely related to vascular endothelial injury. In the scope of our knowledge, this is the first time that IGT has been applied to the field of transplantation.

Macrophage polarization plays an important role in maintaining the homeostasis of the immune microenvironment. In the present study, we found that M1 macrophages infiltrated significantly in ABMR transplanted kidney tissues, both in rat models and humans. These experiments elucidate that the occurrence and development of ABMR are related to M1 macrophage infiltration and that the mechanism may be related to the secretion of pro-inflammatory cytokines and exosomes. In further experiments, we found that M1 secreted a large number of inflammatory factors during polarization. Previous studies have reported that TNF-α and other pro-inflammatory factors are closely related to endothelial injury. We can reasonably suspect that pro-inflammatory factors secreted by M1 cause endothelial cell damage through potential pathways, and then result in ABMR progression. Currently, studies on macrophage polarization in the field of transplantation mainly focus on ischemia-reperfusion injury, and research on rejection, especially ABMR, is still lacking (Zhang et al., 2018; Ye et al., 2020). More importantly, recent studies have pointed out that monocytes and macrophages could be considered as secondary effectors and capable of direct allorecognition, indicating the potential therapeutic target for ABMR treatment (Callemeyn et al., 2022; Lebraud et al., 2022). The concrete molecular mechanism needs to be studied further.

Our *in vitro* experiments indicate that KLF4 had low expression in M1 macrophages while IGT can reverse this trend. We speculated that KLF4 in macrophages could play a protective role by suppressing their M1 polarization. Sharma *et al* demonstrated that KLF4 reduced the secretion of pro-inflammatory cytokines in myeloid cells and thereby constrains atherogenesis (Sharma et al., 2012). This finding is consistent with our research. Xudong Liao et al *believed that KLF4 could* inhibit M1 polarization via the intercept of co-activators required for NF-κB activation (Liao et al., 2011b). These studies indicated that KLF4, as an important regulatory factor, could regulate the polarization process of macrophages.

In recent years, our team has conducted a clinical study in the world for the first time in combination with traditional immunosuppression regimens in kidney transplant recipients. The results showed that the incidence of biopsy-proven acute rejection in the IGT group was significantly lower than that in the control group and no obvious toxic side effects were observed. However, its mechanism of immunosuppression is still unclear, which restricts its further clinical application. In this study, we found that IGT significantly slowed the occurrence of ABMR after surgery, and this process was associated with the targeted inhibition of M1-type macrophages. This result explained part of the mechanism of the effect of IGT, which is conducive to further promoting the clinical application of IGT after kidney transplantation.

Obviously, there are still certain limitations in this study. The mechanism of KLF4 may be further explored. We still need to explore the specific mechanism of the interaction between macrophages and endothelial cells. Furthermore, the LM22 matrix used for the bioinformatics analysis in this study was to explore the immune cell infiltrations, which were originally designed for tumor biology. With regard to the difference in the microenvironment in various pathogenesis, the immune infiltration in renal organs, especially in the renal allograft, should be interpreted with caution.

In conclusion, studies on the rat ABMR model and *in vitro* suggest that IGT may attenuate the progression of ABMR after kidney transplantation by inhibiting KLF4-regulated M1 macrophage polarization. Our study of IGT provided a novel insight into the further exploration of treatment for ABMR.

## Data Availability

The original contributions presented in the study are included in the article/Supplementary Material, further inquiries can be directed to the corresponding authors.

## Supplementary Material

Supplementary Figure S1 IGT showed no effect on M2 macrophage polarization *in vitro*. **(A–D)** mRNA expression of Arg-1 **(A)**, YM1 **(B)**, Fizz1 **(C)**, and MR **(D)** in BMDM after the intervention of IGT (10 μg/ml) for 12 h. A non-parametric test was used for the statistical analysis in this figure.
