# Supplementary figures and images for: Iguratimod Attenuates Macrophage Polarization and Antibody-Mediated Rejection After Renal Transplant by Regulating KLF4
Source: Front Pharmacol. 2022 May 9;13:865363. doi: 10.3389/fphar.2022.865363 (PMC9125033; doi:10.3389/fphar.2022.865363)

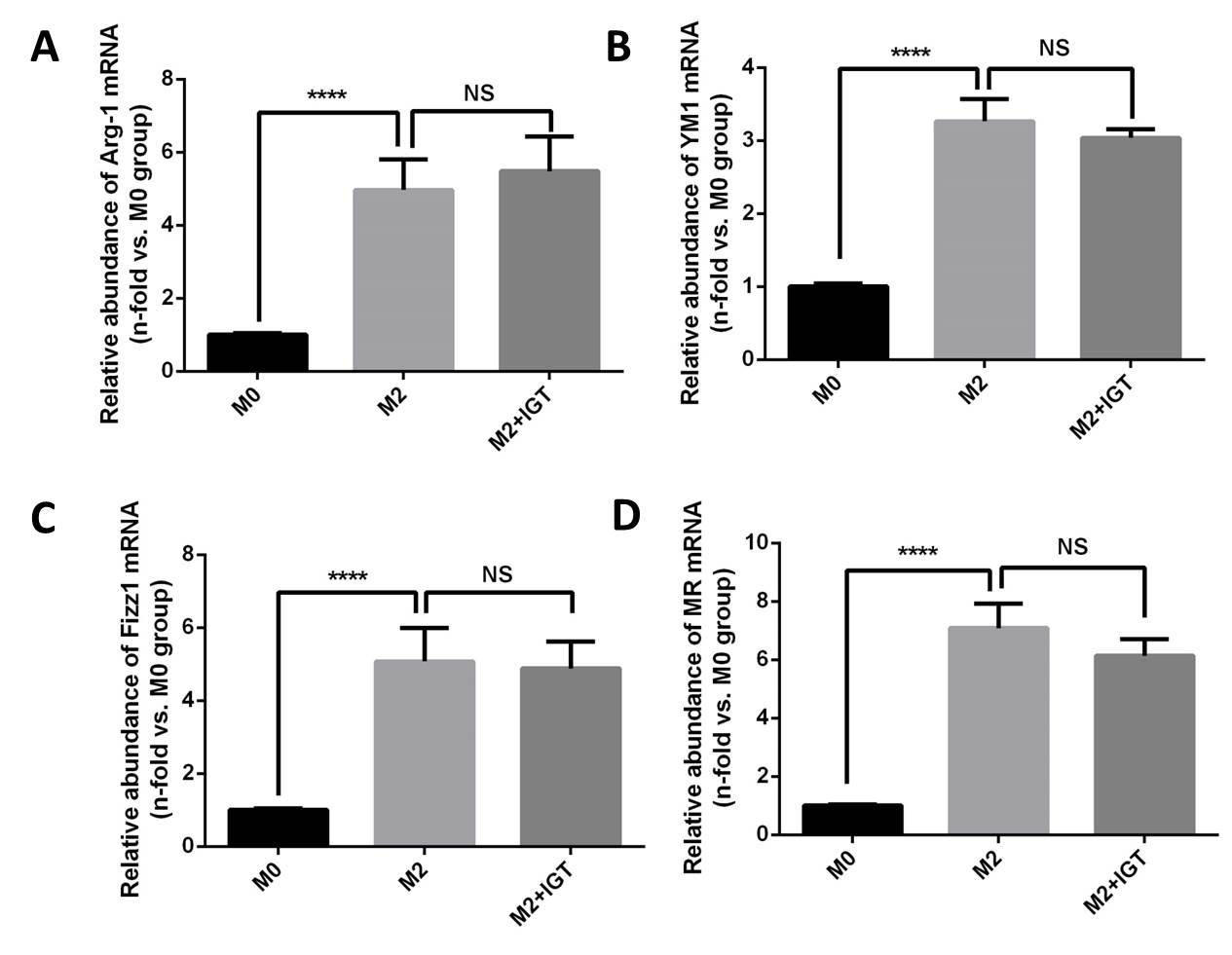

Supplement: Supplementary file 1 [file Image1.JPEG]
